# Novel Aluminum Oxide-Impregnated Carbon Nanotube Membrane for the Removal of Cadmium from Aqueous Solution

**DOI:** 10.3390/ma10101144

**Published:** 2017-09-28

**Authors:** Ihsanullah, Faheemuddin Patel, Majeda Khraisheh, Muataz Ali Atieh, Tahar Laoui

**Affiliations:** 1 Center for Environment and Water (CEW), Research Institute, King Fahd University of Petroleum and Minerals, Dhahran 31261, Saudi Arabia; engr.ihsan.dir@gmail.com; 2 Department of Mechanical Engineering, King Fahd University of Petroleum & Minerals, Dhahran 31261, Saudi Arabia; faheemmp@kfupm.edu.sa; 3 Department of Chemical Engineering, Qatar University, Doha 2713, Qatar; m.khraisheh@qu.edu.qa; 4 College of Science and Engineering, Hamad Bin Khalifa University, Qatar Foundation, Doha 5825, Qatar

**Keywords:** membrane, carbon nanotubes, aluminum oxide, cadmium, toxic metal

## Abstract

An aluminum oxide-impregnated carbon nanotube (CNT-Al_2_O_3_) membrane was developed via a novel approach and used in the removal of toxic metal cadmium ions, Cd(II). The membrane did not require any binder to hold the carbon nanotubes (CNTs) together. Instead, the Al_2_O_3_ particles impregnated on the surface of the CNTs were sintered together during heating at 1400 °C. Impregnated CNTs were characterized using XRD, while the CNT-Al_2_O_3_ membrane was characterized using scanning electron microscopy (SEM). Water flux, contact angle, and porosity measurements were performed on the membrane prior to the Cd(II) ion removal experiment, which was conducted in a specially devised continuous filtration system. The results demonstrated the extreme hydrophilic behavior of the developed membrane, which yielded a high water flux through the membrane. The filtration system removed 84% of the Cd(II) ions at pH 7 using CNT membrane with 10% Al_2_O_3_ loading. A maximum adsorption capacity of 54 mg/g was predicted by the Langmuir isotherm model for the CNT membrane with 10% Al_2_O_3_ loading. This high adsorption capacity indicated that adsorption was the main mechanism involved in the removal of Cd(II) ions.

## 1. Introduction

Cadmium is a well-known highly toxic metal found in drinking water, and is associated with major negative health impacts. The World Health Organization guidelines suggest an allowable limit of cadmium ions, Cd(II), in water of 0.003 mg/L [1]. Cadmium primarily accumulates in the kidneys, and has a relatively long biological half-life of 10 to 35 years in humans [2]. A potential source of cadmium contamination in drinking water is industrial wastewater, such as that produced by the manufacturing processes for smelting, pesticides, fertilizers, dyes, pigments, refining, and textile operations. Cadmium contamination of drinking water might also be caused by the presence of Cd(II) ions as an impurity in the zinc of galvanized pipes and certain metal fittings [3,4].

Various toxic metal decontamination techniques (such as adsorption, precipitation, reduction, ion exchange, precipitation, solvent extraction, electrolytic recovery, and chemical oxidation) have been applied for the removal of toxic metals from water [3,5]. However, the majority of these methods have limited applications due to economical or technical constraints. Water treatment by adsorption offers the most practical and economical treatment alternative. Numerous adsorbents have been used in the removal of metal ions from water, including activated carbon, fly ash, biomaterials, zeolite, recycled alum sludge, algal biomass, peanut hulls, resins, kaolinite, manganese oxides, and carbon nanotubes [6]. However, the majority of the adsorption techniques are batch-level processes and are unable to process large amounts of contaminated water. Carbon nanotube-based novel membranes are a promising candidate for toxic metal removal.

CNTs have attracted considerable attention in recent years as a novel adsorbent for the adsorption of numerous pollutants from water, including toxic metal ions [3,6] and organic chemicals [7,8,9,10]. CNTs have also emerged as an ideal candidate for the synthesis of unique membranes with excellent properties for applications in water treatment [11,12,13]. The friction-less and smooth graphitic walls of CNTs are considered ideal channels for enhanced molecule transport [13,14,15]. CNTs can be used either as fillers to improve the mechanical, electrical, and thermal properties of various polymeric membranes or as a direct filter [16,17,18,19].

Different types of CNT-based membranes have been reported in the published literature. The most common categories include mixed-matrix membranes [16,17,18,19], vertically aligned CNT membranes [20,21], bucky paper membranes [22,23,24], and template-assisted open-ended CNT membranes [25,26].

In this work, a novel approach for the synthesis of CNT membranes is presented using a powder metallurgy technique. To avoid heating at extremely high temperatures (approximately 3000 °C) to bond the CNTs together, aluminum oxide (Al_2_O_3_) particles are impregnated onto the surface of CNTs and used to bond the 3D CNT network together during a sintering process performed at only 1400 °C to produce the CNT membrane. Moreover, surface impregnation of CNTs has been reported to yield an enhanced surface area and better adsorption capacity for the removal of different contaminants from water [27]. CNTs were impregnated via a wet chemistry technique with different loadings of aluminum oxide. Impregnated CNTs were characterized using XRD, and the developed membranes were analyzed to measure the contact angle, porosity, and water flux. The potential of the membrane for cadmium removal was investigated using a continuous flow system. The effect of pH, aluminum oxide loading onto the CNTs, initial cadmium concentration of the solution, and time, on the removal efficiency of Cd(II) ions, was investigated.

## 2. Materials and Methods

### 2.1. Materials

CNTs with a purity >95% were acquired from Chengdu Organic Chemicals Co. Ltd., China (Chengdu, China) with an outside diameter of 10–20 nm and a length of 1–10 μm. Aluminum isopropoxide [C_9_H_21_O_3_Al] (purity ≥ 98%, Sigma Aldrich, Saint Louis, MO, USA) was used as a precursor for aluminum oxide.

### 2.2. Impregnated Aluminum Oxide-Carbon Nanotubes

The surfaces of the CNTs were impregnated with Al_2_O_3_ nanoparticles via the wet impregnation method [28,29]. The loading content of Al_2_O_3_ ranged from 1 to 20% (by weight, wt %). In this method, to prepare 10% Al_2_O_3_ loading, 7.5 g of aluminum isopropoxide [C_9_H_21_O_3_Al] was dissolved in 500 mL of ethanol (98% purity). Nine grams of CNTs were dispersed in a separate 500 mL of ethanol. These mixtures were separately ultrasonicated for 1 h and subsequently mixed together. The mixture was further ultrasonicated for 1 h at ambient temperature, to ensure a homogeneous dispersion of the CNTs. To remove the ethanol solvent, the mixture was stored in an oven overnight at the required temperature. The residue was calcinated in a tube furnace under argon at 350 °C for 4 h. This process results in the attachment of Al_2_O_3_ particles onto the surface of CNTs. A similar procedure was used to prepare CNTs with various Al_2_O_3_ loading percentages (between 1% and 20%).

### 2.3. Membrane Preparation

**Figure 1:**
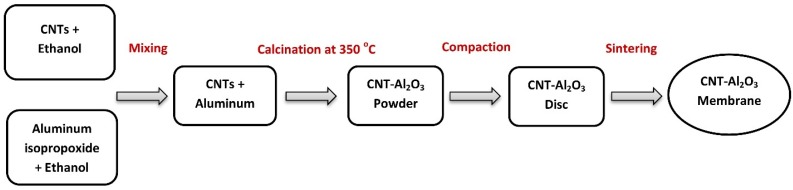
Flowchart for the synthesis of aluminum oxide-impregnated carbon nanotube (CNTs–Al_2_O_3_) membrane.

The Al_2_O_3_-impregnated CNTs, prepared as described in Section 2.1, were uniaxially pressed at 200 MPa compaction pressure using a circular metallic die in a hydraulic bench-type press (4387 N.E.L, Carver, Inc., Wabash, IN, USA). This process resulted in a compact disc (27 mm in diameter and 3 mm thick) containing 1%, 10%, and 20% Al_2_O_3_. The compacted discs were sintered at 1400 °C for 5 h under argon (300–400 mL/min) in a tube furnace (GSL-1700X, MTI Corporation, Richmond, CA, USA). A schematic representation of the synthesis route is shown in Figure 1.

### 2.4. Characterization Analysis of Raw and Impregnated CNTs and Membranes

#### 2.4.1. SEM Analysis

SEM studies of the membranes were performed using field emission scanning electron microscopy (TESCAN MIRA 3 FEG-SEM, TESCAN, Brno-Kohoutovic, Czech Republic).

#### 2.4.2. X-ray Diffraction (XRD)

The XRD patterns for raw and impregnated CNTs were measured at a rate of 1.0°/min in the range of 10°–80° (2α) using an X-ray diffractometer equipped with a Cu Kα radiation source (40 kV, 20 mA).

#### 2.4.3. Porosity Measurement

The dry-wet method [16] was used to determine the porosity of the membranes using Equation (1):

(1) $$\mathrm{Porosity}=\frac{W_{2}-W_{1}}{\rho \cdot V}\times 100\%$$

where *W*_1_ (g) and *W*_2_ (g) are the weights of the dry and wet membranes, respectively; *V* (cm^3^) is the volume of the membrane; and *ρ* (g/cm^3^) is the density of distilled water at ambient temperature. The membrane was immersed in distilled water for 24 h, and its wet weight was measured. The membrane was subsequently dried in an oven at 90 °C for 24 h, and the dry weight of the membrane was measured. The experiment was performed in triplicate, and the data are presented as the mean value of all experiments.

#### 2.4.4. Contact Angle Measurement

A contact angle analyzer (model DM-301, KYOWA, Niiza, Japan) was used to measure the contact angle of the membrane surface and hence the hydrophobicity/hydrophilicity of the membrane.

#### 2.4.5. Zeta Potential Measurement

The zeta potential for a suspension of 0.5 g/L CNTs-Al_2_O_3_ in distilled water was determined using a Malvern ZEN2600 Zetasizer Nano Z (Malvern, Worcestershire, UK) at pH 2.0–10 (adjusted with 0.1 M NaOH or HNO_3_).

### 2.5. Continuous Filtration System

**Figure 2:**
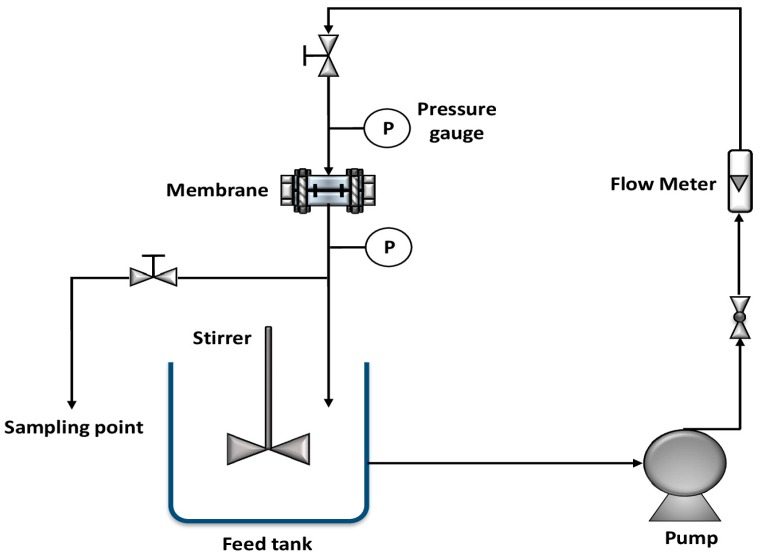
Schematic diagram of the flow loop system.

The main components of the continuous filtration system used in this study (presented in Figure 2) were a membrane cell with an effective surface area of 5.7 cm^2^, a 10 L feed tank, and the required pressure pump and flow meter. The membrane was placed in a circular housing with a mesh underneath it as a support structure to maintain the stability of the membrane during the flow experiments. The pure water flux analysis was performed before the Cd(II) remediation studies were performed. The pure water flux was measured using Equation (2) [16,28,29]:

(2) J=V/(A·t)

where *J* (L·m^−2^·h^−1^) is the water flux, *t* (h) is the time required for permeate water to pass through the membrane, and *V* (L) is the volume of permeate water.

For cadmium removal, the experimental runs began with the circulation of a 1 ppm solution of Cd(II) from the feed tank through the system. An initial volume of approximately 10 L was added to the feed tank, and the pH was adjusted using 1 M NaOH or 1 M HNO_3_, as required. The pressure and flow rate were adjusted to the desired values. Permeate (purified water) passing through the membrane was collected from the sample collection point (shown in Figure 2) at different time intervals using sample bottles with volumes of approximately 20 mL. The effects of the initial concentration, time, pH, and membrane Al_2_O_3_ loading on cadmium removal were studied.

Table 1 shows the experimental conditions for Cd(II) removal using different CNT-Al_2_O_3_ membranes. First, a CNT membrane with 10% Al_2_O_3_ loading was used to determine the optimum pH for the maximum removal of Cd(II). The transmembrane pressure difference and concentration of Cd(II) were held constant during these experiments. The optimum pH (pH = 7) was held constant during the remainder of the experiments, and the effects of Al_2_O_3_ loading and the initial Cd(II) concentration in the solution (water) on the removal efficiency of Cd(II) ions were determined.

### 2.6. Analytical Methods

Inductively coupled plasma mass spectrometry (X-Series 2 Q-ICP-MS, Thermo Fisher Scientific, Waltham, MA, USA) was used to measure the concentration of Cd(II) before and after the experiments.

## 3. Results and Discussion

### 3.1. SEM and EDS Analysis

**Figure 3:**
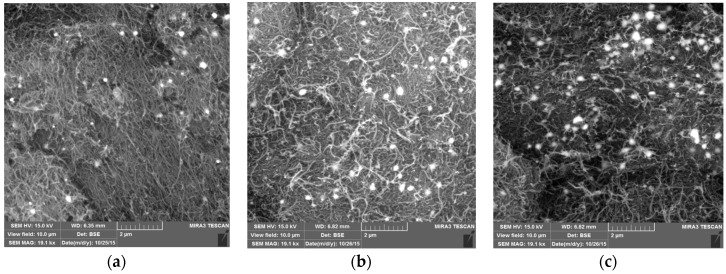
SEM micrographs of CNT membranes impregnated with (**a**) 1%; (**b**) 10%; and (**c**) 20% Al_2_O_3_ particles.

Figure 3 shows the SEM images of the prepared membranes with various Al_2_O_3_ contents. The particles are well dispersed at low Al_2_O_3_ loadings, whereas the particles tend to agglomerate at higher Al_2_O_3_ loadings (i.e., 20% or above).

### 3.2. X-ray Diffraction (XRD)

**Figure 4:**
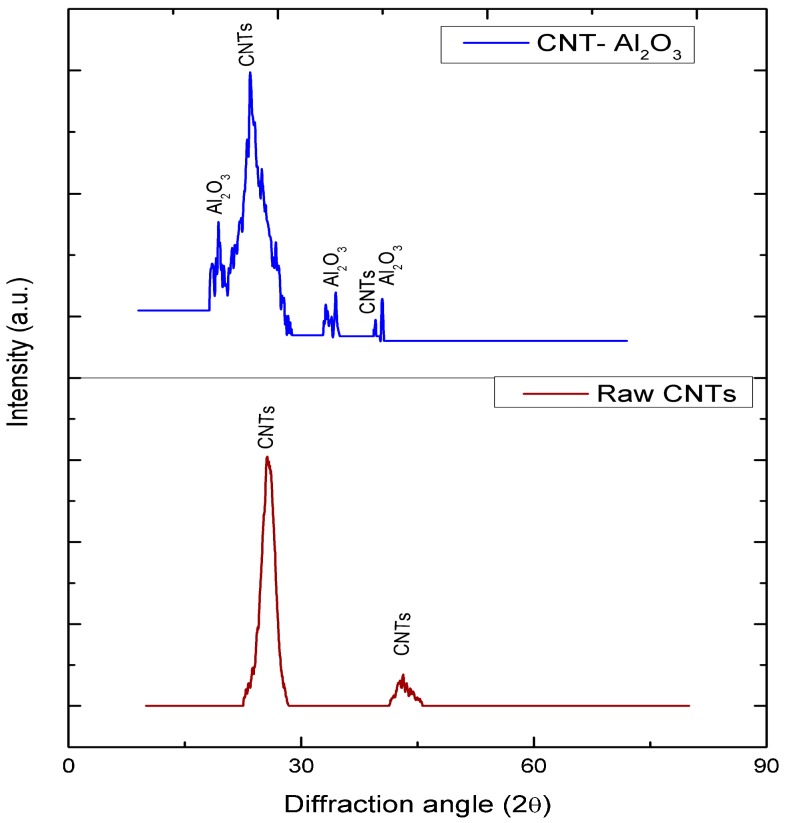
XRD plots of raw and Al_2_O_3_-impregnated CNTs.

Figure 4 displays the XRD patterns of the raw and impregnated CNTs powders. In the XRD pattern of the raw CNTs, the characteristic peaks at 2θ = 27° and 44° correspond to the CNTs. However, the XRD pattern of the impregnated CNTs presents new peaks at 2θ = 17°, 33°, and 40° in addition to the two apparent peaks associated with the CNTs. These peaks correspond to the Al_2_O_3_ nanoparticles and indicate the successful impregnation of Al_2_O_3_ particles onto the surface of the CNTs. The XRD spectrum shows a minor shift of CNTs peaks for the CNT-Al_2_O_3_, possibly due to the residual stresses imposed by the Al_2_O_3_ particles embedded onto the CNTs [30,31].

### 3.3. Measurement of the Zeta Potential and Point of Zero Electric Charge (pH_PZC_)

**Figure 5:**
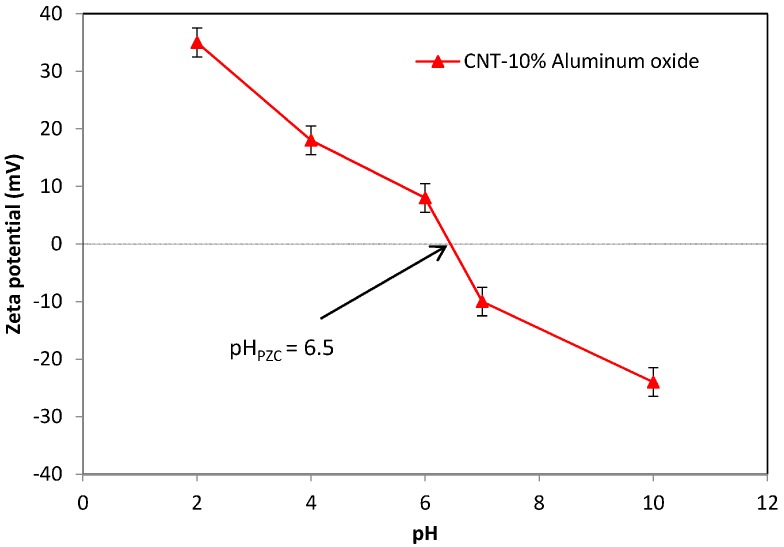
Variation of the zeta potential values with pH for the CNT-Al_2_O_3_ membrane.

The zeta potentials of CNT-10% Al_2_O_3_ in distilled water were determined in a pH range of 2.0–10. As displayed in Figure 5, the surface charge of the membrane surface is positive at pH < 6.5 and negative at pH > 6.5. The zero electric charge (pH_PZC_) value of the membrane was noted at 6.5. The membrane is expected to have a relatively higher removal of Cd(II) ions at pH 7, as discussed in Section 3.6, because of the electrostatic interactions between the negatively charged membrane surface and cationic Cd(II) ions.

### 3.4. Membrane Characterization

#### 3.4.1. Porosity Measurement

**Figure 6:**
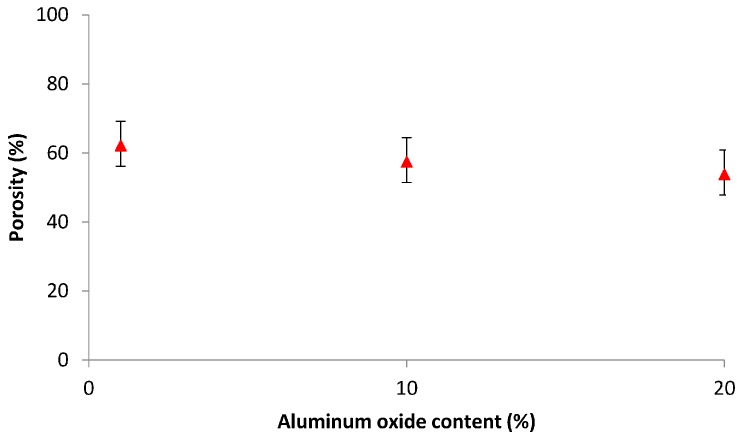
Porosity of the Al_2_O_3_-impregnated CNT membranes.

The dry-wet method was used to determine the porosity of the membranes. Figure 6 displays the porosity versus Al_2_O_3_ loading for loadings of 1 to 20%. Considering the standard deviation reported for the measured porosity values, the variation in porosity with Al_2_O_3_ content is rather minor, implying that the Al_2_O_3_ loading does not have a significant effect on the membrane porosity.

#### 3.4.2. Contact Angle Measurement

**Figure 7:**
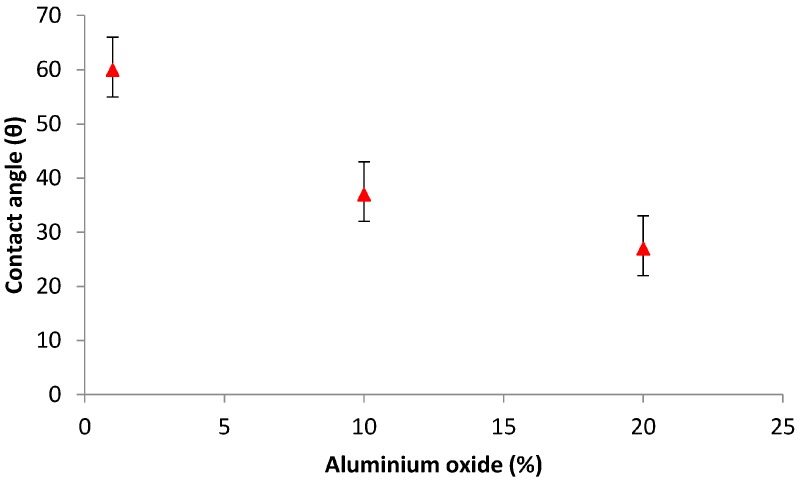
Contact angle of the membrane versus Al_2_O_3_ content.

**Figure 8:**
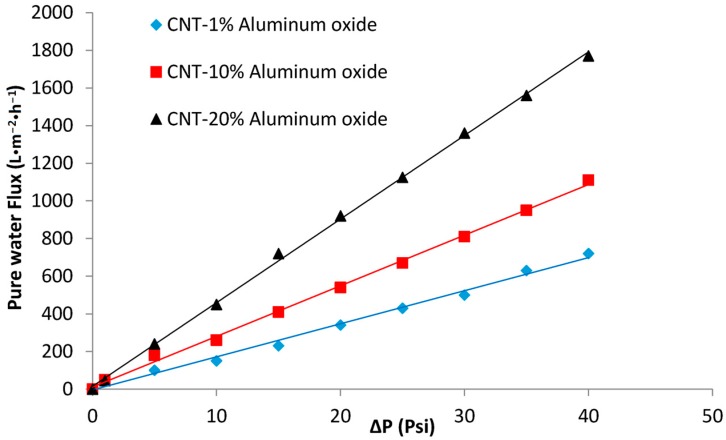
Effect of transmembrane pressure difference on the water flux of the CNT-Al_2_O_3_ membranes.

Contact angle measurement is an index of the hydrophobicity/hydrophilicity of the membrane surface. As shown in Figure 7, the contact angle of the membrane decreased with increasing Al_2_O_3_ content. In other words, the hydrophilicity of the membrane increases with increasing Al_2_O_3_ loading. The decrease in contact angle with increase in Al_2_O_3_ loading might be due to change in membrane pore size due to agglomeration at higher Al_2_O_3_ loading. This agglomeration leads to enhanced water flux (as shown in Figure 8), and hence, the contact angle value is decreased. This hydrophilic nature of the membrane is primarily responsible for the enhanced flux through the membrane [28,29].

### 3.5. Water Flux Measurements: Effect of Transmembrane Pressure Difference and Aluminum Oxide Loading

The effects of transmembrane pressure difference, aluminum oxide loading, and time, on the water flux through the membranes, were studied, as shown in Figure 8. The transmembrane pressure difference was varied from 1 to 40 psi. A nearly linear relationship exists between the pressure and flux for all membranes with different Al_2_O_3_ loadings. The water permeate flux was measured by holding the transmembrane pressure difference constant at 20 psi for 30 min. The permeate flux values for different pressures were obtained using the same procedure. For each reading, the pressure was maintained for 10 min before noting the readings.

Figure 8 illustrates that the permeate water flux increased as the Al_2_O_3_ content increased from 1 to 20%. The increased permeate flux for membranes with high Al_2_O_3_ loading can be explained based on two mechanisms. First, the hydrophilic surface of the membrane at high Al_2_O_3_ loading facilitates the transport of water through the membrane (Figure 7). Second, the agglomeration of Al_2_O_3_ particles (Figure 3c) at high loading (20%) results in the formation of relatively large pores in the membrane, thus contributing to the higher permeate flux.

### 3.6. Cadmium Removal

The Cd(II) ion removal studies were performed in the flow loop system, as shown in Figure 2. The cadmium solution was passed through the CNT-Al_2_O_3_ membrane. Cd(II) ions are retained in the feed side while purified water permeates the membrane. The cadmium removal (R) can be determined using the following equation:

(3) $$R=1-C_{p}/C_{f}$$

where *C_p_* and *C_f_* are the concentrations of the solute in the permeate and feed, respectively.

#### 3.6.1. Effect of Feed pH

**Figure 9:**
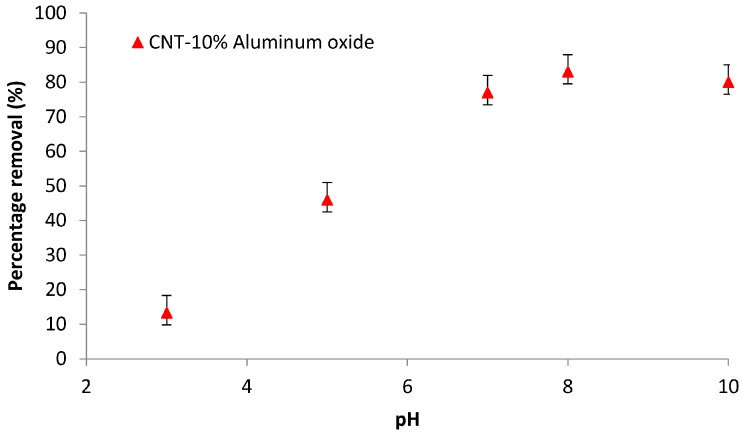
Effect of pH on the percentage removal of Cd(II) ions by CNT-10% Al_2_O_3_ membrane (initial concentration = 1 ppm, time = 3 h).

Solution pH is an important parameter that determines the removal of toxic metal by carbon-based materials. The Cd(II)removal experiments were performed for the membrane with 10% Al_2_O_3_ loading in the pH range of 3–10 (experimental set 1). The results of the analysis are shown in Figure 9.

Cadmium species are present in deionized (DI) water in the form of Cd^2+^, Cd(OH)^+^, and Cd(OH)_2(s)_ [32,33]. At pH < 8, the dominant cadmium species is Cd^2+^ in the form of complex [Cd(H_2_O)_6_]^2+^ [34]. The pH_PZC_ value for Al_2_O_3_-doped CNTs is pH 6.5, as shown in Figure 4. This value demonstrates that Al_2_O_3_ is basic in DI water [27]. At pH < pH_PZC_, the membrane surface is positively charged, and repulsion exists between the Cd(II) ions and surface, causing a low removal rate for Cd(II) ions. In addition, competition between H^+^ and Cd^2+^ ions for the active sites decreases the Cd(II) ions adsorption rate. At pH > pH_PZC_, the surface of the membrane becomes more negatively charged, and thus, additional Cd(II) ions are attracted to the surface, due to electrostatic interactions.

A maximum removal of 84% was observed at pH 8. However, cadmium might have precipitated as Cd(OH)_2_ at pH > 8, as reported elsewhere [35,36], and the removal might be due to both adsorption and precipitation. Therefore, pH 7 was used in all experiments as an optimum value to avoid the precipitation of cadmium ions. Moreover, at pH > 8, the concentration of Cd(II) ions is low, and the predominant ions are ${\mathrm{HCO}}_{3}^{-}$. The repulsion between the negatively charged surface of the membrane at pH > pH_PZC_, and the ${\mathrm{HCO}}_{3}^{-}$ ions, causes a decrease in the removal of cadmium ions. In addition to electrostatic interactions, the van der Waals interactions occurring between the cadmium ions and carbon atoms could also induce the adsorption of cadmium ions [27,37].

#### 3.6.2. Effect of Time

**Figure 10:**
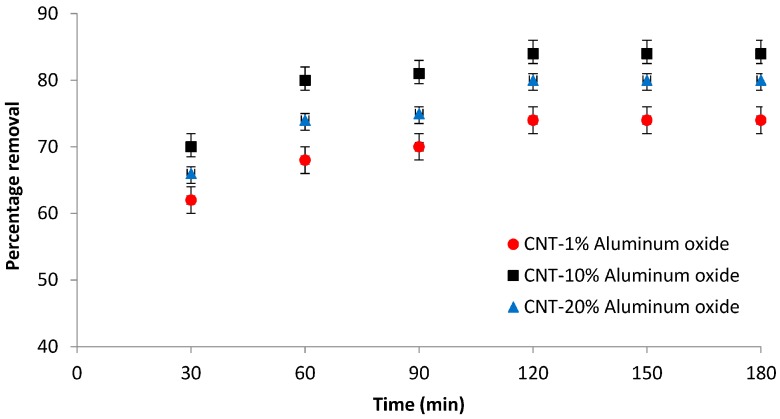
Effect of time on the percentage removal of cadmium ions by the CNT-Al_2_O_3_ membranes (initial concentration = 1 ppm, pH = 7).

To study the effect of time on the removal of Cd(II) ions, the experiments were performed at constant pH 7, and an initial concentration of 1 ppm. The samples were collected every 30 min and analyzed. The results of the analysis are presented in Figure 10. The percentage removal of Cd(II) increases with time until 2 h of operation, after which no significant increase in removal was observed. Equilibrium was reached within nearly 2 h for all membranes. The maximum removal rate of 84% was achieved with the CNT-10% Al_2_O_3_ membrane. Membranes with 1% and 20% Al_2_O_3_ loadings achieved Cd(II) removal rates of 80% and 74%, respectively, under similar conditions.

The relatively higher removal of Cd(II) ions by the CNT-10% Al_2_O_3_ membrane might be due to the greater number of adsorption sites than the membrane with 1% Al_2_O_3_ loading. The relatively lower removal of Cd(II) ions by the membrane with 20% Al_2_O_3_ loading could be attributed to the agglomeration of Al_2_O_3_ particles at higher loading i.e., 20% Al_2_O_3_ (as discussed in Section 3.1 (Figure 3c)). This agglomeration might create large pores in the membrane that leads to poor separation of Cd(II) ions.

#### 3.6.3. Effect of the Initial Concentration

**Figure 11:**
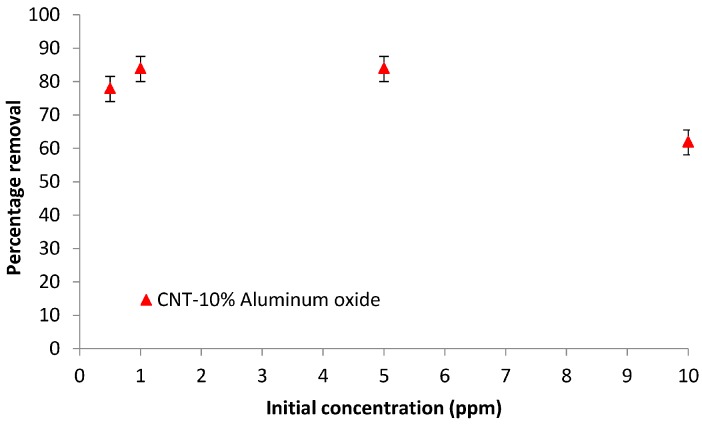
Effect of the initial concentration on the percentage removal of cadmium ions by the CNT-10% Al_2_O_3_ membrane (contact time = 2 h, pH = 7).

Figure 11 presents the effect of the initial concentration of the solution on the percentage removal of Cd(II) ions. The initial concentration was varied from 0.5 to 10 ppm, and the other experimental parameters were pH 6, a contact time of 2 h, and a transmembrane pressure difference of 15 psi.

The removal increased slightly from 78 to 84%, as the concentration increased from 0.5 to 1 ppm, and remained nearly constant until approximately 5 ppm. At an initial concentration of 0.5 ppm, the membrane still had available adsorption sites and did not reach saturation; thus, more Cd(II) ions could be adsorbed. The percentage removal remained constant as the concentration was increased from 1 to 5 ppm. However, as the concentration increased further beyond 5 ppm, the removal decreased slightly, likely because all available adsorption sites were covered by the Cd(II) ions and the membrane had reached its adsorption equilibrium limit. The membrane was effective in removing a low concentration of Cd(II) ions.

#### 3.6.4. Adsorption Isotherms

**Figure 12:**
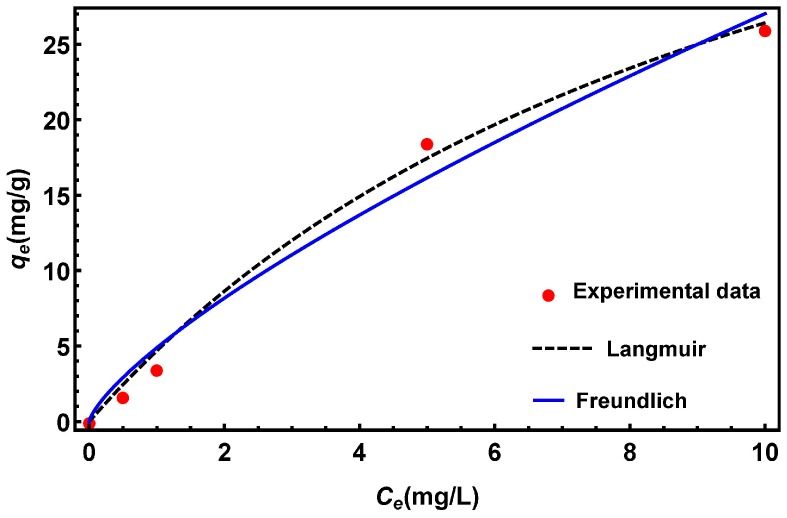
Langmuir and Freundlich adsorption isotherm model fits for the removal of cadmium ions by the CNT-10% Al_2_O_3_ membrane.

The nonlinear forms of the Langmuir and Freundlich adsorption isotherms for the adsorption of cadmium ions on the CNT-Al_2_O_3_ membrane surface are presented in Figure 12. Representative equations and the results of the analysis are summarized in Table 2.

In the Langmuir model, *q_e_* (mg/g) represents the concentration of adsorbate on the surface of adsorbent, *C_e_* (mg/L) indicates the concentration of adsorbate in solution when equilibrium was reached, *q_m_* (mg/g) is the maximum adsorption capacity, and *K_L_* is the Langmuir adsorption equilibrium constant (L/mg). In the Freundlich isotherm model, *K_F_* (mg/g)·(L/mg)^1/*n*^ and *n* (dimensionless) are Freundlich constants. Mathematica version 10 (Wolfram 2015, Long Hanborough Oxfordshire, UK) was used to plot the isotherm data and determine the values of various parameters.

Both models can describe the experimental data satisfactorily, but the correlation coefficient (R^2^) value for the Langmuir isotherm model is slightly higher than that for the Freundlich isotherm model.

### 3.7. Mechanism of Cadmium Ion Removal by the CNT-10% Al_2_O_3_ Membrane

**Figure 13:**
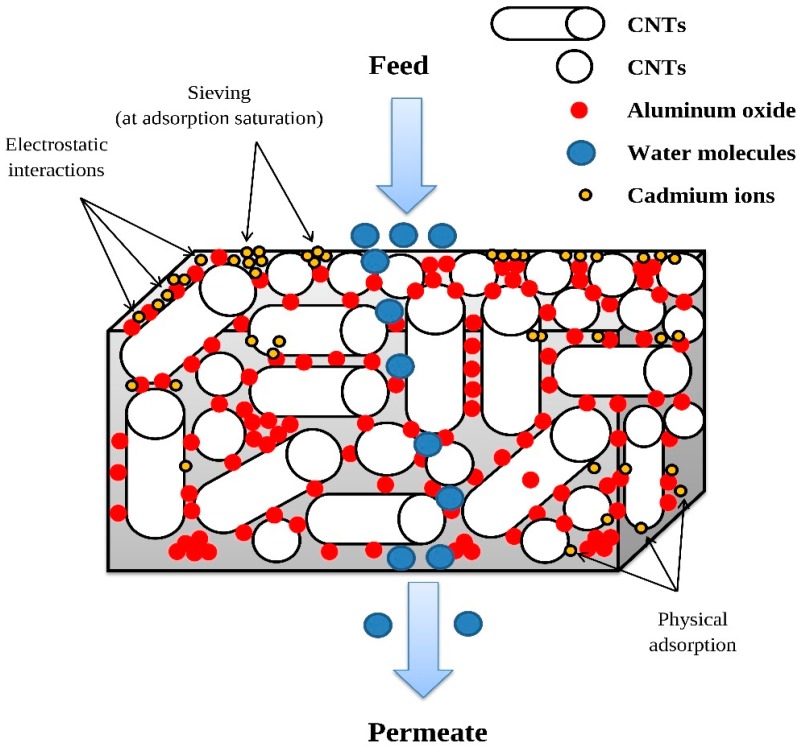
Possible mechanisms of Cd(II) ion interactions with the CNT-Al_2_O_3_ membrane.

The possible mechanism underlying Cd(II) ion interactions with the CNT-Al_2_O_3_ membrane is presented in Figure 13. As discussed in Section 3.6.1, the dominant cadmium species in deionized (DI) water is Cd(II), or Cd^2+^, in the form of complex [Cd(H_2_O)_6_]^2+^ at pH 7, used as an optimum value in all experiments. When pH < pH_PZC_, the membrane surface is positively charged, and the low removal of Cd(II) ions can be attributed to electrostatic repulsions between the Cd(II) ions and the surface of the CNT-Al_2_O_3_ membrane. Similarly, the higher removal of Cd(II) ions at pH > pH_PZC_ might be due to the strong electrostatic interactions between the negatively charged CNT-Al_2_O_3_ membrane surface and the cationic Cd(II) ions [27].

This observation suggests that electrostatic interaction is the main mechanism involved in the sorption of Cd(II) ions onto the CNT-Al_2_O_3_ membrane surface. In addition to electrostatic interaction, Cd(II) ions might also adsorb on the surface of CNT-Al_2_O_3_ membrane due to van der Waals interactions (physical adsorption) occurring between Cd(II) ions and carbon atoms in the CNT-Al_2_O_3_ composite. At the adsorption saturation of the membrane surface and internal structure, certain pores among the CNTs might be covered by Cd(II) ions, thus blocking further ions from passing through and acting as sieves (size exclusion).

### 3.8. Comparative Analysis

The adsorption capacity and removal efficiency of Cd(II) ions from water for the membrane developed in this study are compared to those of similar studies reported in the literature in Table 3. The estimated maximum Cd(II) adsorption capacity of the CNT-10% Al_2_O_3_ membrane is 54.42 mg/g, which is higher than those of related adsorbents (used in batch experiments), such as raw CNTs (1.661 mg/g) [38], acid-modified CNTs (4.35 mg/g) [3], ethylenediamine-functionalized multi-walled carbon nanotubes (MWCNTs) (25.70 mg/g) [33] and nano-alumina on single-walled carbon nanotube (SWCNTs) (2.18 mg/g) [39]. This result suggests that the CNT-Al_2_O_3_ membrane is effective in removing low concentrations of Cd(II) ions from water.

## 4. Conclusions

A novel approach was developed to synthesize an aluminum oxide-impregnated CNT membrane. No binder was used in the membrane synthesis; instead, aluminum oxide particles served as a binder to hold the 3D CNT network together. The membrane surface demonstrated extreme hydrophilic behavior and yielded a high water flux. The membrane was able to remove low concentrations of Cd(II) ions from aqueous solution. The removal was affected by the aluminum oxide loading, initial cadmium solution concentration, pH, and time. The maximum Cd(II) removal of 84% was obtained at pH 7 and an initial concentration of 1 ppm using the CNT membrane with 10% Al_2_O_3_ loading and 2 h of operation. Membranes with 1% and 20% Al_2_O_3_ loadings were able to remove 80% and 74% of Cd(II) ions, respectively, under similar experimental conditions. These results suggest that the CNT-Al_2_O_3_ membrane can be effectively used in a continuous filtration system for the removal of Cd(II) ions.

## Figures and Tables

**Table 1:** Experimental matrix of parameters used in the removal of cadmium with the developed membranes.

Experimental Set	Al_2_O_3_ Loading (wt %) in the Membrane	Transmembrane Pressure Difference (psi)	pH of the Solution	Initial Concentration of Cd(II) (ppm)
1	CNT-10% Al_2_O	15	3, 5, 7, 8, 10	1
2	CNT-1% Al_2_O_3_	15	7	0.5, 1, 5, 10
3	CNT-10% Al_2_O_3_	15	7	0.5, 1, 5, 10
4	CNT-20% Al_2_O_3_	15	7	0.5, 1, 5, 10

**Table 2:** Langmuir and Freundlich isotherm model parameters for the adsorption of Cd(II) ions on the CNT-10% Al_2_O_3_ membrane.

Model	Parameters	CNT-10% Al_2_O_3_ Membrane
Langmuir ($q_{e}=\frac{q_{m\;K_{LC_{e}}}}{1+K_{LC_{e}}}$)	*K_L_* (L/mg)	0.09
	*q_m_* (mg/g)	54.42
	R^2^	0.997
Freundlich ($q_{e}=K_{F}\cdot Ce^{1/n}$)	*K_F_* (mg/g)·(L/mg)^1/*n*^	4.89
	N	1.35
	R^2^	0.99

**Table 3:** Comparative analysis of the adsorption capacity and removal efficiency of Cd(II) ion removal.

Adsorbent	Experimental Conditions	Percentage Removal	Adsorption Capacity (mg/g)	Reference
As-grown CNTs	pH 5.5, initial concentration = 4 mg/L	-	1.1	[2]
H_2_O_2_ oxidized CNTs	pH 5.5, initial concentration = 4 mg/L	-	2.6	[2]
HNO_3_ oxidized CNTs	pH 5.5, initial concentration = 4 mg/L	-	5.1	[2]
Acid-modified CNTs	pH 7, initial concentration = 1 ppm	93	4.35	[3]
Raw CNTs	pH 7, initial concentration = 1 ppm	27	1.661	[38]
Ethylenediamine-functionalized MWCNTs	pH 8, initial concentration = 5 mg/L	-	25.70	[33]
SWCNTs	pH 8	-	1.97	[39]
Nano-alumina/SWCNTs	pH 8	-	2.18	[39]
SWCNTs-COOH	pH 5, initial concentration = 20 mg/L	69.97	55.89	[40]
CNT-10% Al_2_O_3_ membrane	pH 7, initial concentration = 1 mg/L, contact time = 2 h	84	54.42	This study
